# Antimicrobial photodynamic therapy on teeth with molar incisor hypomineralization—controlled clinical trial

**DOI:** 10.1097/MD.0000000000017355

**Published:** 2019-09-27

**Authors:** Letícia Diniz Santos Vieira, Marco Aurelio Benini Paschoal, Pamella de Barros Motta, Elza Padilha Ferri, Caroline Diniz Pagani Vieira Ribeiro, Lourdes Aparecida Martins dos Santos-Pinto, Lara Jansiski Motta, Marcela Letícia Leal Gonçalves, Anna Carolina Ratto Tempestini Horliana, Kristianne Porta Santos Fernandes, Raquel Agnelli Mesquita Ferrari, Alexandre Melo Deana, Sandra Kalil Bussadori

**Affiliations:** a FACIPLAC, Brasília, DF; b Department of Pediatric Dentistry and Orthodontics, Federal University of Minas Gerais, Belo Horizonte, MG; c Biophotonics Applied to Health Sciences, University Nove de Julho, SP; d Uniceplac Brasília; e University Julio de Mesquita Filho, Araraquara, SP, Brazil.

**Keywords:** ART, laser therapy, MIH, PDT

## Abstract

**Background:**

Molar incisor hypomineralization (MIH) is a change in the formation of dental enamel of systemic origin that affects at least one of the first 4 permanent molars and usually affects incisors. During the eruption, the affected surfaces tend to fracture, exposing the dentin, which causes excessive sensitivity in addition to making the region very susceptible to the appearance of carious lesions. The objective of this research will be to evaluate the clinical effect of antimicrobial photodynamic therapy (aPDT) in permanent teeth with severe and sensitive MIH.

**Methods:**

The methodology will be based on the selection of patients from 6 to 12 years of age with permanent molar teeth, randomly divided in 2 groups. The selected teeth should have MIH on the occlusal surface, indicated for clinical restorative treatment. In Group 1, aPDT will be applied for the treatment of infected dentin. Afterward, the teeth will be restored with high viscosity glass ionomer cement. In Group 2, the removal of the softened dentin around the side walls of the cavity with sharp dentine curettes and posterior restoration with high viscosity glass ionomer cement will be performed. All patients will have clinical and radiographic follow-up with a time interval of 6 and 12 months. The data obtained will be submitted to descriptive statistical analysis to evaluate the association of categorical variables. Chi-square test and Fisher exact test will be applied, to analyze the correlation between the continuous variables, Pearson correlation test will be applied. For the analysis of dentin density in the scanned radiographic images and the microbiological results for colony-forming units, ANOVA and Kruskal–Wallis will be applied.

**Discussion:**

Often in the presence of severe MIH, the presence of dentin sensitivity is also associated with caries lesion, making it even more necessary to respect the principles of minimal intervention.

**Trial registration:**

NCT03904641.

## 1 Introduction

The formation of dental enamel occurs through mineral deposition by cells called ameloblasts. Ameloblasts are extremely sensitive and can be affected by systemic factors, which often lead to defects in enamel formation. Depending on the stage of development in which the ameloblasts are affected, different types of changes may occur, such as hypomeneralization.^[1]^ Molar incisor hypomineralization (MIH) is a change in the formation of dental enamel of systemic origin that affects at least one of the first 4 permanent molars and usually affects incisors.^[2,3]^ In the presence of this alteration, the enamel is fragile, porous, and shows areas of white, yellow or brown color, frequently asymmetric. During eruption, the affected surfaces tend to fracture, exposing the dentin, which causes excessive sensitivity, in addition to making the region very susceptible to the appearance of carious lesions.^[4,5]^

A new diagnostic method was developed by Cabral et al in 2019, based not only on the presence, but also on the severity of the MIH. This method is called MIH severity scoring system (MIH-SSS) and is based on the following codes: (0) normal enamel translucency, no enamel opacity; (1) the presence of white/creamy enamel opacity; (2), the presence of yellow/brown opacity; (3) posteruptive breakdown (PEB) restricted to the enamel associated with white/creamy opacity; (4) PEB restricted to the enamel associated with yellow/brown opacity; (5) PEB exposing dentin, the dentin is hard; (6) PEB exposing dentin, the dentin is soft; (7) atypical restoration without marginal defect; (8) atypical restoration with marginal defect; and (9) tooth extracted due to MIH.^[6]^

Due to the clinical characteristics of the teeth with MIH, they become niches of biofilm accumulation. As they also have sensitivity, hygiene is often inadequate, favoring once again the appearance of caries and the eventual involvement of the pulp.^[7]^ Treatment alternatives range from oral hygiene guidance and prevention to restoration, endodontics or extraction, according to the degree of severity of the alteration. The principles of minimal intervention should be taken into account when planning the treatment.^[8]^

Minimal intervention associated with the knowledge of cariology allowed for profound changes in the paradigm of the restorative treatment of caries, since the most striking change involved the principle of maximum preservation of healthy dental structures capable of remineralization.^[9,10]^

Currently, the partial removal of caries, aiming at maintaining pulp integrity, has been considered the therapy of choice in the treatment of deep lesions, provided that certain diagnostic principles are respected.^[10–12]^ In minimally invasive clinical treatment, through different procedures,^[13,14]^ it is recommended to remove the more superficial layer of infected dentin, which is irreversibly denatured and not remineralizable.^[12–14]^ In contemporary dentistry, infected dentin is described with the term “soft dentine” as a concept that defines a softened, often moist tissue that can be easily removed with a curette.^[15,16]^ In the concept of minimal intervention, the selective removal of soft dentine is recommended, that is, it is completely removed from the lateral walls of the cavity, but it is maintained in the pulp wall, in order to avoid pulpal exposure and try to preserve the health of the pulp.^[17]^

The most profound or affected dentin is reversibly denatured, poorly or uninfected, remineralizable and should therefore be preserved. The selective removal of the carious tissue is done, leaving only 1 layer of dentin (a little more hardened) in the deeper portion of the lesion.^[9,17,18]^ Thus, several aspects are important for indicating and performing these procedures, such as a reaction capacity of the dental–pulp complex in front of caries lesion, the initial diagnosis, differentiation of the existing dentin layers into a carious lesion, and the best material indicated for application on the remaining dentin.^[19–21]^

The idea of chemical-mechanical removal of caries consists of the application of a chemical that aims to soften the carious tissue that is removed with a noncutting curette.^[10]^ Papacárie is a gel composed of papain and chloramine. ^[9,22,23]^ Chloramine has properties related to disinfection.^[24]^ Papain is an enzyme similar to human pepsin, which acts as an anti-inflammatory debrisant, not damaging healthy tissue and accelerating the cicatricial process, has bactericidal, bacteriostatic and anti-inflammatory action, and softens the carious tissue facilitating its removal.^[21,24–27]^ By adding methylene blue to the Papacárie formula it can be used in antimicrobial photodynamic therapy (aPDT).^[26,28]^

Antimicrobial photodynamic therapy consists of the use of a photosensitizer that is activated by light and, in the presence of oxygen, produces reactive oxygen species capable of promoting bacterial death.^[29–34]^

## 2 Justification

Studies show that aPDT has been widely used in dentistry,^[31–33,35]^ including in the treatment of caries lesions.^[32]^ However, there are few studies in the literature that report the use of aPDT in teeth with hypomineralization. The literature shows that the aPDT with the use of Papacárie associated with methylene blue presents satisfactory results in the treatment of carious lesions in deciduous teeth, with regard to the microbial reduction.^[36]^

**Figure 1:**
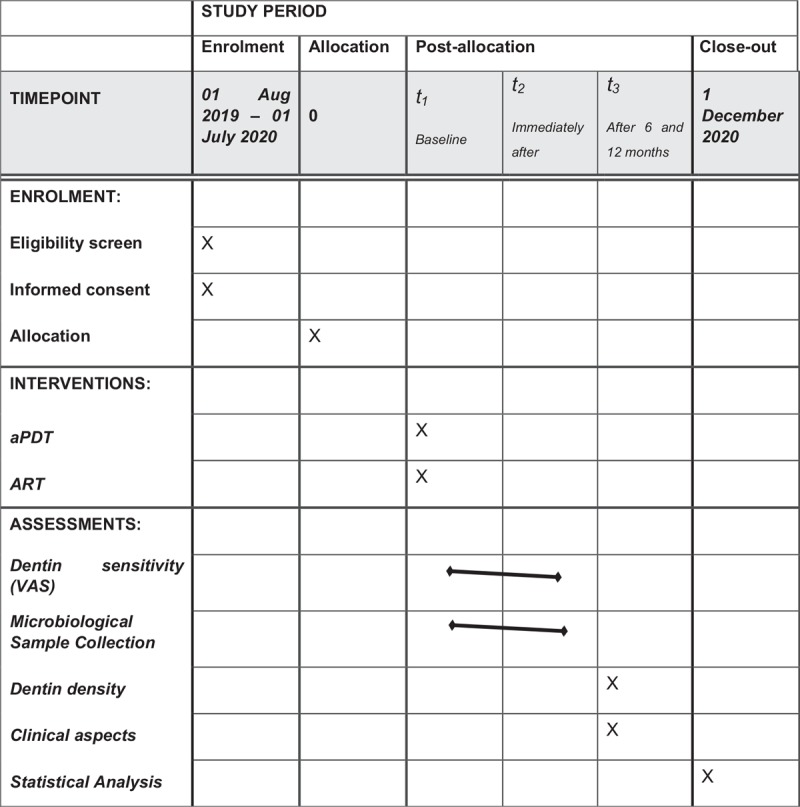
Schedule of enrolment, interventions, and assessments of the present study.

The justification for performing aPDT with Papacárie is to promote a conservative and minimally invasive treatment, reducing the risk of pulp exposure in permanent teeth with MIH (Fig. 1).

## 3 Methods/design

### 3.1 Objectives

#### 3.1.1 General objectives

To investigate the application of Photodynamic Therapy (PDT) in permanent teeth with severe molar incisor hypomineralization, with painful sensitivity, associated with the presence of caries lesion. PapacárieMblue,^[28]^ modified with the addition of methylene blue as a photosensitizer, in conjunction with the low-power laser for laser therapy will be used for desensitization and decontamination of the cavities.

#### 3.1.2 Specific objectives

To compare the use of aPDT with ART.

Hypothesis (H1): The application of Photodynamic Therapy (PDT) in permanent teeth with severe molar incisor hypomineralization, with painful sensitivity, associated with the presence of caries lesion is effective for desensitization and decontamination of the cavities.

Hypothesis (H0): The application of Photodynamic Therapy (PDT) reduces the number of viable bacteria, but is not effective in the treatment of painful sensitivity.

#### 3.1.3 Type of study

A randomized and blind controlled clinical trial will be conducted.

#### 3.1.4 Participants

Forty-eight teeth of healthy 6 to 12-year-old children of both genders, without distinction of race or ethnicity, will be selected at the clinic of the dentistry course of Nove de Julho University (São Paulo, Brazil).

#### 3.1.5 Inclusion criteria

1. Healthy children, without systemic alterations;
2. Collaborative children;
3. Present 1 permanent molar with the code 6 on MIH severity scoring system (MIH-SSS), active and acute caries lesion in dentin, not exceeding 2/3 and involving only the occlusal, with direct vision and access, without clinical and radiographic signs and symptoms of pulp involvement.

#### 3.1.6 Exclusion criteria

1. Child with systemic impairment;
2. Non-cooperative behavior;
3. Carious lesion of Class II, III, IV, or V type of Black;
4. Clinically: carious lesion involving enamel, deficient restorations, small carious lesions in dentin (without access to hand excavators), occult caries lesions, clinical sign and/or symptom of pulp involvement, clinical impossibility of restoration;
5. Radiographically: evidence of pulpal involvement, carious lesion extending beyond two-thirds of the dentin.

#### 3.1.7 Interventions

##### 3.1.7.1 Group 1

1. Initial interproximal radiography;
2. Relative insulation (lip retractor, cotton roller, and saliva sucker);
3. Taking the VAS scale for pain as responded by the volunteer;
4. Microbiological outlet with an otoscopic curette to standardize the volume of carious tissue;
5. Application—(PapacarieMblue), leave action for 20 seconds and remove the softened carious tissue just around the sidewalls of the cavity with blunt dentin spoons. Wait another 3 minutes to act on the blue dye present in the gel composition;
6. Radiate with the low power laser (single point), following the parameters described in item 3.1.10;
7. Clean with cotton ball and water;
8. Second microbiological outlet with curette of remaining dentin tissue;
9. Clinical evaluation by inspection of remaining dentin texture with rounded probe exploratory probe;
10. Clean with cotton ball and water again;
11. Sealing the cavity with glass ionomer cement (Riva Self Cure/HV - SDI);
12. Taking VAS for Pain Responded by Volunteer;
13. Preservation;
14. Radiographic control 6 and 12 months.

##### 3.1.7.2 Group 2

1. Initial interproximal radiography;
2. Relative insulation (lip retractor, cotton roller, and saliva sucker);
3. Microbiological outlet with an otoscopic curette to standardize the volume of carious tissue;
4. Removal of the softened dentin dentine around the lateral walls of the cavity with sharp dentine curettes # 17;
5. Second microbiological outlet with dentin curette;
6. Clinical evaluation by inspecting the texture of the remaining dentin with exploratory catheter;
7. Clean with cotton ball and water;
8. Sealing the cavity with glass ionomer cement (Riva Self Cure/HV - SDI);
9. Preservation;
10. Radiographic control 6 and 12 months.

#### 3.1.8 Sample size

The sample calculation was performed based on previous studies presented in the literature,^[37]^ considering the expected difference and standard deviation, weighting the CFU (bacterial colony forming units) variable. For the statistical calculation, paired samples were considered: α = 1.96 (5%); β = 0.84 (20%); test power 80%. The minimum number for this clinical trial per group was 16 teeth. 
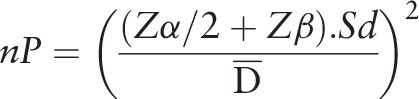


**Figure:**
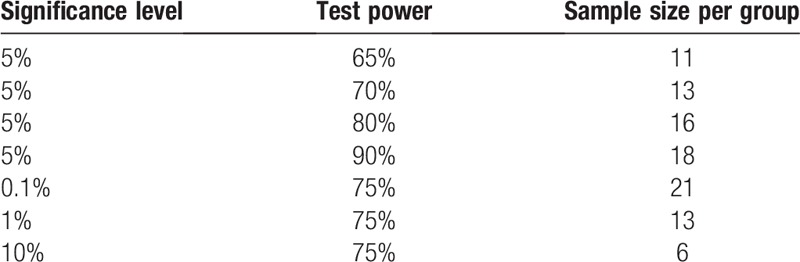


#### 3.1.9 Experimental groups

Forty-eight teeth of healthy 6 to 12-year-old children of both genders, without distinction of race or ethnicity in the clinic of the dentistry course of Nove de Julho University (São Paulo, Brazil) will be select.

The participants will be allocated in the experimental groups as follows:

Group 1—PDT + atraumatic restaurative treatment (ART) (n = 24): for the PDT the PapacarieMblue (Fórmula e Ação) will be used. It will be left for 5 minutes in the cavity. The carious tissue will be removed and the application of the PapacarieMblue will be repeated. The tissue will then be irradiated in a single spot with a red laser, of wavelength of 660 nm, for 60 seconds, with the energy of 6J. These parameters were also applied in the vestibular root of the teeth, to try and prevent sensitivity. Laser parameters can be found in item 3.1.10. Selective removal of carious tissue will be made with a curette, followed by cavity cleaning and restoration with Riva Self Cure/HV – SDI.

Group 2—Atraumatic restaurative treatment (ART) (n = 24): Removal of infected dentin with a curette only, followed by cavity cleaning and restoration with Riva Self Cure/HV – SDI.

#### 3.1.10 Use of low-level laser for antimicrobial photodynamic therapy

For PDT, a low power laser will be used. The equipment Therapy XT emits red and infrared laser light. The apparatus will be calibrated according to the previous mathematical calculations to determine the parameters for antimicrobial photodynamic therapy. The laser will be applied in continuous mode, 100 mW, 2471 cm^2^, 148 J/cm^2^, irradiance 2471 mW/cm^2^, λ= 660 nm, and 6J, 60 seconds per spot. Session will be performed at the dental clinic, and at the moment of application only the volunteer to be treated and the responsible professional (operator) will be present. Specific eyewear shall be used. The active tip of the laser will be coated with clear disposable plastic (avoiding cross contamination). Based on the study by Guglielmi et al in 2011,^[15]^ the laser application will be punctual (single point) and in contact with the cavity.

#### 3.1.11 Procedures

All treatments will be performed by a single operator who will be calibrated during an initial phase of the survey. An operator will perform the treatment and a pretrained examiner will assess whether removal of carious tissue did not reach the pulp. The examiner will not know to which group the participants belong. This pretrained examiner will evaluate all cavities after the respective interventions and is responsible for attesting the presence of soft dentine. The operator and the examiner must agree on the conclusion of the removal of carious dentin. This procedure will also be made in the clinical and radiographic evaluations by the previously calibrated examiners (calculation of Kappa to measure interobserver agreement above 85%).

The clinical evaluations of the complete removal of the carious tissue, microbiological and radiographic will be made by 2 blind examiners, who will not know the treatment performed on each of the teeth.

#### 3.1.12 Evaluation of dentin sensitivity—primary outcome

Before removal of the carious dentin, Group 1 and Group 2 volunteers will respond to the Visual Analogue Scale (VAS),^[38]^ which will obey the following protocol: gauze isolation of neighboring teeth; light air jet in the tooth with MIH for 2 seconds; sensitivity evaluation through the VAS scale. This sequence will be repeated at the end of the procedure, after insertion of the glass ionomer cement.

#### 3.1.13 Microbiological evaluation

Prior to the removal, the soft dentine of each selected tooth will be collected. The removed tissue will be standardized using a No. 2 Meyhoefer curette and will be deposited in a test tube containing 3.8 mL of Transport Medium-Phosphate Buffered Saline (PBS, Labcenter, São Paulo). The biological material will be deposited in the transport flask containing glass beads by shaking the tube at maximum speed in a Vortex apparatus for homogenization of the biological material for 30 seconds. After stoving at 37°C for 30 minutes to liquefy the gelatin from the medium, the dental biofilm will be dispersed into the transport flask containing glass beads by shaking the tube shaker at full speed for 30 seconds.

Subsequently, biological material will be serially diluted in the order of 10^1^ to 10^6^ in peptone water and inoculated into culture media in Petri dishes. Aliquots of dilutions 10^4^, 10^5^, and 10^6^ will be seeded on the surface of Brucella Agar (Difco, Kansas City, USA) plus sheep defibrinated blood (50 mL/L), hemin (Inlab, São Paulo, Brazil) (5 mg/mL), and menadione (Inlab) (10 mg/mL) for the determination of the total viable microorganisms. Aliquots of dilutions 10^3^ and 10^4^ will be seeded in MitisSalivarius agar medium (Difco) to determine the total number of streptococci (S). Biofilm aliquots without dilution and at dilutions 10^1^ and 10^2^ will be seeded in MitisSalivarius Agar medium plus debacitracin to determine the streptococci mutans (SM) population.^[39]^ 100 μL aliquots of each dilution will be seeded on the agar surface and spread with the aid of a Drigalski loop.

In order to verify the presence of lactobacilli (LB), aliquots (100 μL) without dilution and in the dilution 10^2^ will be inoculated in depth (Agarplate Rogosa SL (Difco).^[39]^ Brucella Agar plates will be incubated in anaerobic chamber (PLAS by LABS, LANSING, MICH., USA) at 37°C for 7 days. The plates of MitisSalivarius Agar and MitisSalivarius Bacitracin will be incubated in 10% CO2 (ShelLab CO2 greenhouse, mod. 2123, Oregon) at 37°C for 48 hours. Plates containing Rogosa Agar will be incubated in 10% CO2 at 37°C for 72 hours.^[39]^ After the incubation, the characteristic colonies will be counted in each plate, observed using a stereoscopic microscope with 10-fold increase, in the dilution that presented between 30 and 300 colonies per plate. All procedures will be performed in duplicate and the average counts will be calculated.

The results will be presented in CFU of SM and LB and in percentage of streptococci (% S/TM), streptococci mutans (% SM/TM), and lactobacilli (% LB/TM) in relation to total viable microorganisms. For SM, the proportion of total streptococci (% SM/S)^[15]^ will also be calculated. Immediately after removal of the carious tissue, the remaining dentin will be collected using the Meyhoefer auricular No. 17 curette and the same procedures described above will be adopted.

#### 3.1.14 Radiographic evaluation

Interproximal radiographs will be performed initially, aiming at assessing the presence of caries on the proximal surface (adjacent surfaces), radiolucency compatible with carious lesion in dentin, deep caries and to rule out the possibility of pulp involvement. Hereafter, the controls will be in 6 and 12, to analyze the optical density and visual clinical interpretation of the remaining dentin, even as the evaluation with the radiographic subtraction technique.^[40]^ All images will be made with KODAK Double Films. To better understand the application of the radiographic subtraction technique, the 4 radiographs will be compared and must be taken with the minimum geometrical distortion, which is achieved by individualizing prefabricated radiographic film positioners by means of occlusal records with self-curing acrylic resin, specific for each patient. These should be stored for subsequent radiographic shots.

The radiographic processing should be standardized, such as the time of radiographic exposure, kilovoltage and mili-amperage of the apparatus, and type of film, in order to obtain density and contrast as “identical” as possible between the images.^[40–42]^ The standardization of radiographic processing will be used and the same time of revealing, intermediate washing, fixation, and final washing, in all periods, should be the same. The chemicals used will be Kodak. The images obtained radiographically in the different intervals will be scanned for analysis of the density difference between them. To do so, the program Imagelab 2.3^[41]^ will be used. Thus, it is expected that the density of the remaining dentin will follow the variations in optical density, so they will be evaluated in the time intervals between 6 and 12 months after therapeutic application in all groups. The objective will be to quantitatively determine the gray tones of the affected dentin region just below the restoration in glass ionomer and compare the density of the remaining dentin in both groups.^[41–44]^

#### 3.1.15 Evaluation of time required for procedure

The necessary time for each procedure will be measured using a stop watch (Kenko, Hong Kong) in minutes and seconds from the onset of treatment until complete curettage of soft dentine and restoration. Time will be recorded on a specific chart for analysis.

#### 3.1.16 Clinical evaluation

The criteria used for clinical evaluation are the retention of the material in the cavity and the presence of secondary caries. The evaluation scores will be evaluated based on the results of studies by other authors (Frencken et al).^[11]^ Digital photographs of the restorations will also be performed to complement the elements of clinical and radiographic information. The visual demonstration can contribute to the necessary clarifications and make the discussion and documentation of the cases in this study more efficient. Therefore, digital photographs (Canon Sx500 IS camera), of all the teeth, will be performed in the 2 groups, before and after the interventions. The teeth will be classified as follows

0 = present, without defect.

1. = present, small defects in the margin less than 0.5 mm deep, no need for repair.

2. = present, small defects in the margin of 0.5 to 1 mm depth, needs repair.

3. = present, gross defects in the margin of 1 mm or more in depth, needs repair.

4. = absent, restoration almost / completely lost, needs treatment.

5. = absent, other treatment was performed for any other reason.

6. = tooth missing due to any reason.

7. = present, surface wear less than 0.5 mm, no need for replacement.

8. = present, surface wear greater than 0.5 mm, need replacement.

9. = impossible to diagnose.

#### 3.1.17 Statistical analysis

Data will be analyzed statistically using different tests, considering the level of significance of 5%. The data obtained will be submitted to descriptive statistical analysis to evaluate the association of categorical variables. Chi-square test and Fisher exact test will be applied, and to analyze the correlation between the continuous variables, Pearson correlation test will be applied. For the analysis of dentin density in the scanned radiographic images and the microbiological results for colony-forming units, ANOVA and Kruskal–Wallis will be applied.

## 4 Discussion

Often in the presence of MIH, the occurrence of dentin sensitivity is also associated with caries lesion, making it even more necessary to respect the principles of minimal intervention. According to some authors in situations of deep lesions associated with MIH (especially at risk of pulpal exposure) the selective removal of soft dentine should be applied, that is, removal of this soft tissue only from the surrounding walls, maintaining the soft dentine of the wall pulpar.^[16]^ Since contaminated tissue is kept inside the cavity in these situations, the application of decontamination methods such as PTT is necessary. Some studies suggest that the use of the PapaMblue gel in aPDT is effective, in addition to producing more toxic oxygen species than the free methylene blue.^[36]^

## Author contributions

**Conceptualization:** Letícia Diniz Santos Vieira, Marco Aurelio Benini Paschoal, Pamella de Barros Motta, Elza Padilha Ferri, Sandra Kalil Bussadori.

**Data curation:** Letícia Diniz Santos Vieira, Marco Aurelio Benini Paschoal, Caroline Diniz Pagani Vieira Ribeiro.

**Formal analysis:** Alexandre Melo Deana Melo Deana.

**Investigation:** Letícia Diniz Santos Vieira, Marcela Letícia Leal Gonçalves, Sandra Kalil Bussadori.

**Methodology:** Marco Aurelio Benini Paschoal, Lara Jansiski Motta, Marcela Letícia Leal Gonçalves, Sandra Kalil Bussadori.

**Project administration:** Lara Jansiski Motta.

**Software:** Alexandre Melo Deana Melo Deana.

**Supervision:** Lourdes Aparecida Martins dos Santos-Pinto, Anna Carolina Ratto Tempestini Horliana, Kristianne Porta Santos Fernandes, Raquel Agnelli Mesquita Ferrari.

**Validation:** Anna Carolina Ratto Tempestini Horliana, Kristianne Porta Santos Fernandes.

**Visualization:** Lourdes Aparecida Martins dos Santos-Pinto, Marcela Letícia Leal Gonçalves, Kristianne Porta Santos Fernandes.

**Writing – original draft:** Letícia Diniz Santos Vieira, Sandra Kalil Bussadori.

**Writing – review & editing:** Pamella de Barros Motta, Elza Padilha Ferri, Lourdes Aparecida Martins dos Santos-Pinto, Lara Jansiski Motta, Anna Carolina Ratto Tempestini Horliana, Raquel Agnelli Mesquita Ferrari, Sandra Kalil Bussadori.
